# ASSOCIATION BETWEEN PRE-HIP FRACTURE DEPRESSION AND DAYS AT HOME AFTER FRACTURE AMONG MEDICARE BENEFICIARIES

**DOI:** 10.1093/geroni/igae098.1158

**Published:** 2024-12-31

**Authors:** Rhea Mehta, Denise Orwig, Chixiang Chen, Yu Dong, Michelle Shardell, Takashi Yamashita, Jason Falvey

**Affiliations:** University of Maryland Baltimore, Baltimore, Maryland, United States; University of Maryland School of Medicine, Baltimore, Maryland, United States; University of Maryland School of Medicine, Baltimore, Maryland, United States; University of Maryland School of Medicine, Baltimore, Maryland, United States; University of Maryland School of Medicine, Baltimore, Maryland, United States; University of Maryland Baltimore County, Baltimore, Maryland, United States; University of Maryland School of Medicine, Baltimore, Maryland, United States

## Abstract

Hip fracture and depression are important public health issues among older adults. Though historically more common in females, hip fracture cases are projected to increase among males, who often experience greater depression severity. Days at home (DAH), a patient-centered metric reflecting fracture recovery, can inform post-surgical treatments for older adults. The association between pre-fracture depression and DAH among hip fracture survivors and potential sex differences remains unexplored. Our cohort included 63,618 community-dwelling Medicare fee-for-service beneficiaries, aged 65+, who underwent hip fracture surgery between 2010 and 2017. Generalized Estimating Equations assessed longitudinal associations between pre-fracture depression at hospital admission and total DAH over 12 months post-discharge, adjusting for covariates. Sex-by-depression interactions were also included, and multiple models were built to identify factors that may explain the primary association. There was no difference in DAH between beneficiaries by depression status in the fully adjusted model. After adjusting for demographic factors (age and sex) and social determinants of health (race, Medicaid dual eligibility, and poverty), those with depression spent 11 fewer average DAH compared to counterparts without depression (incident rate ratio [IRR] = 0.92; 95% CI = 0.91, 0.93; p<.0001). The association was attenuated and not significant after additional adjustment for medical complexities, specifically comorbidities, and facility and geographic factors. The sex-by-depression interaction term was not statistically significant (p=0.38). The association between pre-existing depression and DAH among Medicare beneficiaries with hip fracture underscores the need for early recognition and secondary prevention of depressive symptoms during acute care, which may inform hip fracture treatment.

